# Using group concept mapping to co-create a research agenda with the homeless community in Rhode Island

**DOI:** 10.1186/s40900-026-00850-3

**Published:** 2026-02-23

**Authors:** Megan Smith, Elizabeth Goulart, David Araujo

**Affiliations:** Rhode Island College, Providence, RI USA

**Keywords:** Homelessness, Community-based participatory research, Group concept mapping, Patient and public involvement

## Abstract

**Background:**

Homelessness is indisputably a pressing social concern in the United States today, with deleterious impacts on both individual and population health. However, policy development often excludes the voices of those directly affected, leading to strategies that fail to meet their needs. This study was grounded in the principle that people experiencing a phenomenon have the clearest view of its causes and solutions. We used a community-based participatory research design, group concept mapping, to partner with the homeless community in Rhode Island to understand what is needed to effectively address this crisis.

**Methods:**

People with current or recent experiences of homelessness were eligible to participate, and recruitment occurred primarily by word-of-mouth between partners (participants) and their peers. The research involved 43 partners in step one, where they responded to an open-ended prompt, “When we understand ______ better, the homelessness crisis will be better,” to generate 25 statements. In step two, 50 partners sorted these statements based on similarity and rated them on a scale of 1 (not at all important) to 4 (very important). The data were analyzed using multidimensional scaling and hierarchical cluster analysis to visualize the interconnections and group the statements. The research team reached consensus on a five-cluster solution.

**Results:**

The five core clusters were: immediate and basic needs (average rating 3.66), longer-term needs (3.48), interactions with authority (3.45), core factors of well-being (3.11), and the social experience of homelessness (3.11). The single highest-rated topic was housing availability, affordability, and stability (3.93). Crucially, partners conceptually grouped mental health and substance use care within the *interactions with authority* cluster, suggesting that behavioral health services are often mandated rather than voluntarily sought in the community.

**Conclusions:**

This study confirms that community-based participatory research is feasible and effective in developing a research and action roadmap with people experiencing unsheltered homelessness. The results suggest the necessity of adapting healthcare and social service systems to be more responsive to the needs of the homeless community, including by emphasizing the importance of voluntary treatment models. The process centered partners’ perspectives, validating their lived expertise and inviting their continued collaboration in future research.

## Abstract (de)

This study used a community-based participatory research approach to learn directly from people experiencing homelessness in Rhode Island about how to address this crisis. Based on the understanding that those experiencing a problem have the clearest view of its causes and solutions, the co-authors partnered with community members to develop a roadmap for action and future research. First, 43 partners completed the statement: “When we understand ______ better, the homelessness crisis will be better,” resulting in 25 topics. Then, 50 partners grouped these topics based on similarity and rated them by importance. Partners often did these activities together. Partners found the crisis to be complex, highlighting both specific needs and broad social issues. They clustered their priorities into five areas: short-term needs, long-term needs, interactions with authority, core needs, and the social experience of homelessness. The highest-rated individual element was housing availability, affordability, and stability. The study’s findings can help us design more responsive health care and social service systems. For example, partners frequently connected mental health and substance use treatment with policing and courts. This suggests the importance of identifying pathways for people to connect to these services through voluntary rather than mandatory means. This study demonstrates the value and feasibility of centering the perspectives of people experiencing homelessness in research about how to address it.

## Background

It is well-established that homelessness – and in particular unsheltered homelessness – is deleterious for those experiencing it [1] and impacts the broader community [2]. For the individuals directly impacted, this includes social stigmatization [3], poor access to health care [4], and high disease burden [5]. The combined weight of these factors contributes to premature mortality: people experiencing homelessness die some 20–25 years younger than their housed counterparts [6]. How the impacts on the broader community are conceptualized varies by social and political perspective, with some highlighting consequences such as petty crime (e.g., trespassing, shoplifting, selling or sharing drugs) [7] and the negative economic impacts of encampments [8] and activities like panhandling (begging) [9]. Another point of view argues that the social harm comes from not having the strengths, capacities, and perspectives of people experiencing homelessness folded into our broader community fabric. This position asserts that integrating these individuals into our neighborhoods would improve them for all [10].

Despite broad recognition of the problem of homelessness, there is not consensus about how to best address it, with policymakers’ perspectives ranging from the low-barrier and voluntary [11] to the carceral and compulsory [12]. The former end of the spectrum includes housing first programs that provide apartments with no conditions beyond those of a standard lease coupled with voluntary wraparound services [13], and harm reduction programs that partner with individuals and communities directly impacted by substance use to distribute materials including clean needles and the overdose reversal drug naloxone [14]. The latter end includes required drug treatment in residential substance use treatment facilities, often imposed as a condition of criminal sentencing [15], and the use of civil commitment laws to involuntarily hospitalize people experiencing homelessness and severe mental illness [16].

Furthermore, the voices of those directly impacted are often not substantively considered when developing policy and practice interventions, causing these strategies to misalign with the realities, needs, and goals of those ostensibly to be served [17]. For example, shelter and housing options often misalign with individuals’ family compositions and life logistics. This includes restrictions on couples staying together, prohibitions on pets, curfews that do not allow for overnight work, and the threat of expulsion if substance use is discovered [18]. With respect to healthcare access, this includes administrative dismissals for missed outpatient appointments and the withholding of follow-up care for self-managed (against medical advice) hospital discharges, both of which are often related to the very same complicated circumstances for which the individual is seeking support [19, 20].

Among those studies that have gathered data directly from people experiencing homelessness, recurrent themes include both individuals’ capacity to meet their basic needs through creative means and the deficits in the resources necessary for them to do so [21]. Multiple studies highlight the ingenuity of people experiencing homelessness in finding ways to make money (e.g., through panhandling, scrapping metal, shoplifting, selling drugs, doing sex work) [22, 23] and finding places to stay (e.g., reinforcing tents, locating abandoned structures, identifying unutilized land) [24]. However, these strategies have associated limitations: the amount of money earned is often not adequate to cover daily expenses, and the physical configuration of the space is often insufficient to fully protect against the weather. Furthermore, many of tactics carry a risk of criminal legal system involvement: it is illegal in the United States to shoplift, sell drugs, and do many forms of sex work, and people are subject to removal and arrest for trespassing on land to which they do not have a legal claim [25, 26].

While housing was certainly identified as a critical need by the participants in these studies, so too were safety, education, employment, and medical care [27]. Safety included both physical safety on the street [18] and psychological safety with service providers and in residential and care settings [28]. With respect to education and employment, people experiencing homelessness identified the material and psychological benefits of these activities [29], while also recognizing the logistical barriers [30]. Participants identified a need for medical care in a range of settings and contexts (outpatient and inpatient; for acute and chronic concerns), and highlighted the importance of accessible, timely, and person-centered care [31]. The perception of the quality of services – particularly of behavioral health services – was also variable [27]. While some participants noted the positive impacts of practical supports and help with social reintegration [32], others found services to be overly rigid and prescriptive and insufficiently trauma-informed [33]. Studies have emphasized the social experience of being othered and dehumanized [34] and how homelessness itself is traumatic [35]. Participants in these studies also highlighted the interconnectedness of these elements, for example how health is impacted by the daily experience of homelessness [34].

This study was grounded in the concepts of epistemic authority [36] – that those experiencing a phenomenon have the clearest vantage point on its causes and solutions – and structural competence: that we need to listen critically and develop a working understanding of communities’ day-to-day realities in order to adapt our work to be relevant to their needs and goals [37]. To operationalize these conceptual elements, we used a community-based participatory research (CBPR) design, group concept mapping (GCM). CBPR, a broad term that encompasses multiple philosophies and modalities that share an inductive approach to knowledge generation, sees community participants as full partners in the research process [38]. Its core principles include the amplification of community strengths, collaboration, co-learning and capacity building, knowledge sharing, and longitudinal commitment [39]. GCM provides a structured way to gather and visually represent community members’ co-generated perspectives on a particular topic. The research participants, hereafter referred to as partners to better reflect their integral engagement with the study, define both the scope of the topic and its distinct elements, building upon this to identify interconnections and priorities [40]. By involving partners in multiple steps of idea generation, data collection, and data analysis, GCM provides a process by which to crystallize community perspectives in alignment with the philosophy and practice of CBPR [41]. GCM has been used with vulnerable populations including fragile families [42], people re-entering the community after incarceration [43], and neighborhoods addressing their social determinants of health [44]. To the authors’ knowledge, there is only one published study that uses GCM with people experiencing homelessness, and this study focused specifically on barriers to diabetes care [45].

While existing studies highlight some issues of importance to the homeless community, they are sparse and several are dated, which is of particular importance given (a) the place-to-place variability in experiences of homelessness and (b) its changing nature given larger phenomena including the worsening housing crisis [46] and changes to the illicit drug supply [47]. Furthermore, the focus of these studies has been at the level of the individual participant (e.g., What resources have they utilized and how useful have they been?) rather than at the level of the community (e.g., What resources, programs, or practice adaptations would benefit a particular subset of the homeless community?). This does not make space for participants in these studies to offer their perspective on shared experiences and needs.

We addressed these gaps in the literature by using GCM to partner with the homeless community in Rhode Island to identify what should be prioritized in addressing the homelessness crisis, to our knowledge the first time that this approach was used with this group for this purpose. We aimed to learn with and from this community itself (a) what its members believe is needed to address the crisis of homelessness, and (b) how important these elements are to them and how they see them as interconnected. Through a multi-stage process, partners responded to an open-ended prompt, *When we understand ______ better*,* the homelessness crisis will be better*, then rated and sorted the topics their peers generated based on perceived importance and interconnections. Consistent with the tenets of CBPR, partners were invited to participate as peer researchers during both stages, conducting the study activities with those in their social network.

## Methods

In using GCM as a CBPR methodology with the unsheltered homeless community, we were intentional in adapting it in ways that maintained fidelity to its core tenets, particularly its focus on centering community input at each step while minimizing “menu-setting” or steering from the researchers. We also proactively considered how we could leverage partners’ strengths, particularly their strong connections with peers. To accomplish this, it was critical for us to develop a research design that was ethical and practical, consider our own roles and positionalities, be intentional about partner inclusion and recruitment, systematize data collection and analysis, and proactively address potential ethical concerns. We sought to incorporate lived expertise from multiple perspectives throughout the research process: this included both having a co-author with lived experience with homelessness and incorporating partners in the data collection, analysis, and dissemination.

### Research design

Consistent with Kane & Trochim’s (2007) approach, our GCM process involved four steps. Steps one and two explicitly involved gathering the community perspective. Step one generated ideas by asking partners to respond to a broad prompt: *When we understand ______ better*,* the homelessness crisis will be better*. This statement was intentionally phrased to include the word “we” to invite respondents to think about answers relevant to their peers and others as well as to themselves. The co-authors asked people with current or recent experience with homelessness in Rhode Island to fill in this blank as many times as they chose, and the researchers then distilled these responses into a list of 25 topic statements comprising the most common responses. In the second step, which involved the community in analyzing the data from step one, we asked currently or formerly homeless individuals (the same study population as step one – either the same or different specific people) to rate these topic statements based on perceived importance and to sort them into groups based on similarity of meaning.

We incorporated partners as peer researchers in steps one and two: once a person completed the study activity, they were then invited to complete it with a peer in the role of researcher. In step three, the co-authors used multidimensional scaling and hierarchical cluster analysis to generate a concept map visualizing partners’ associations among these topics. The fourth step, sharing the clusters and priority items back with the homeless community and identifying next steps, is still in progress. We are working with a partner – an individual who participated in steps one and two of the process – to develop an illustrated infographic of the key findings to disseminate, which will include an invitation to partner for subsequent studies (described in the discussion).

The co-authors – who facilitated the collaborations with partners – consist of the principal investigator (PI) (an early-career academic who has done homelessness outreach but who has not herself experienced homelessness), and two research assistants (RAs) (one of whom has recent personal experience with unsheltered homelessness, one of whom is a Master of Social Work student without personal experience with homelessness). The PI first met the RA with lived experience on outreach while he was homeless and in talking with him learned that he was curious about research and consulting work. Once housed, he also expressed a desire to find ways to stay connected with his peers who were still experiencing homelessness. Because of this stated interest, he was the first person to whom the PI reached when funding for this project was secured. The RA who is an MSW student responded to an email sent by the PI inviting students interested in participating in this research to contact her.

At the inception and throughout the process, the co-authors engaged in reciprocal teaching and learning. The PI shared information about research ethics (e.g., informed consent, managing dual relationships) and about data coding and analysis (e.g., how to format demographic data; how to use SAS). The RA with lived experience shared about daily life in the encampments and suggested dates, times, and locations for data collection based on this knowledge. He also contributed his firsthand perspective when interpreting partners’ responses. The RA who is an MSW student shared her expertise in instructional design. As the person least familiar with homelessness at the outset, she also asked curious questions that helped us to check our assumptions and clearly articulate our thinking.

The study was approved by Rhode Island College’s Institutional Review Board and was funded by a grant through the Rhode Island College Committee on Faculty Scholarship.

### Partners

A total of 43 individuals participated in step one of the study and 50 participated in step two, exceeding our target of 40 for each step. This sample size allowed for a range of partner viewpoints and demographics while remaining feasible for the research timeline, the co-authors’ capacity, and funding parameters. Inclusion criteria for those with whom we partnered were (1) currently experiencing homelessness or acute housing insecurity in Rhode Island or having experienced this within the past 12 months, (2) being at least 18 years of age, and (3) ability to communicate in English. We used convenience and snowball sampling for recruitment. While we did post flyers in places that people experiencing homelessness regularly visit, the majority of partners heard about the study through word of mouth from peers. Welcoming partners to recruit people with whom they live and interact strengthened the study by incorporating their self-defined communities.

We reviewed the informed consent script with partners verbally, giving them the option to read along from a printed copy. We sought verbal consent rather than written consent because a signed consent form would have been the only identifying document retained. The co-authors verbally consented all partners regardless of whether the partner did the study activity with the co-authors or with a peer. Partners were paid $20 cash at the start of the study activity. Partners were paid another $30 to complete the study activity as a peer researcher; the partner who completed the study activity with the peer researcher was also paid $20. The ethical considerations of this payment model are discussed below.

### Data collection

We conducted the vast majority of study activities in or near tent encampments. While the study design was created to allow for asynchronous completion of the peer researcher component (the peer researcher could take a packet with detailed instructions and the study materials, complete them with a peer, and then later return them to the co-authors), in practice most partners preferred to complete the activity together in pairs or as a group. Partners regularly coached one another, giving examples of a particular topic or offering alternative wording to describe a concept. We did not discourage collaboration and information sharing. We believe that partners talking with one another as part of the study activities enhanced the responses shared, particularly given that the stated intention was to elicit input on community needs and goals rather than solely individualized ones.

Materials included printed copies of each of the study worksheets described below, as well as pens and plastic envelopes that served both to protect study materials from the weather and to provide a writing surface. Partners could choose to complete the worksheets themselves or to say their answers to a co-author or peer, who wrote them. For step one, partners were given a worksheet with the statement *When we understand ______ better*,* the homelessness crisis will be better* and ten lines on it and were asked to give as many responses as they wanted.

For step two, partners were given a set of 25 index cards and three worksheets. Each index card had one of the statements generated during step one on one side, and a number on the other side. They were given the instructions, “Sort these topics into piles based on how similar they seem to one another. You can have as few or as many piles as you want. All cards must go into a pile. Each card can only go in one pile. One-card piles are okay.” Each partner did their own sorting, and documented in the grid on the first worksheet how they sorted the cards by recording the numbers. On the second worksheet, they rated how important they thought each statement was on a scale of 1 (not at all important) to 4 (very important). On this same sheet they stated which topic they thought was most important and why. The third worksheet asked demographic and homelessness history questions. As with step one, the co-authors asked partners whether they preferred to write their own answers or have one of the co-authors or a peer do so.

### Data analysis techniques

**Fig. 1:**
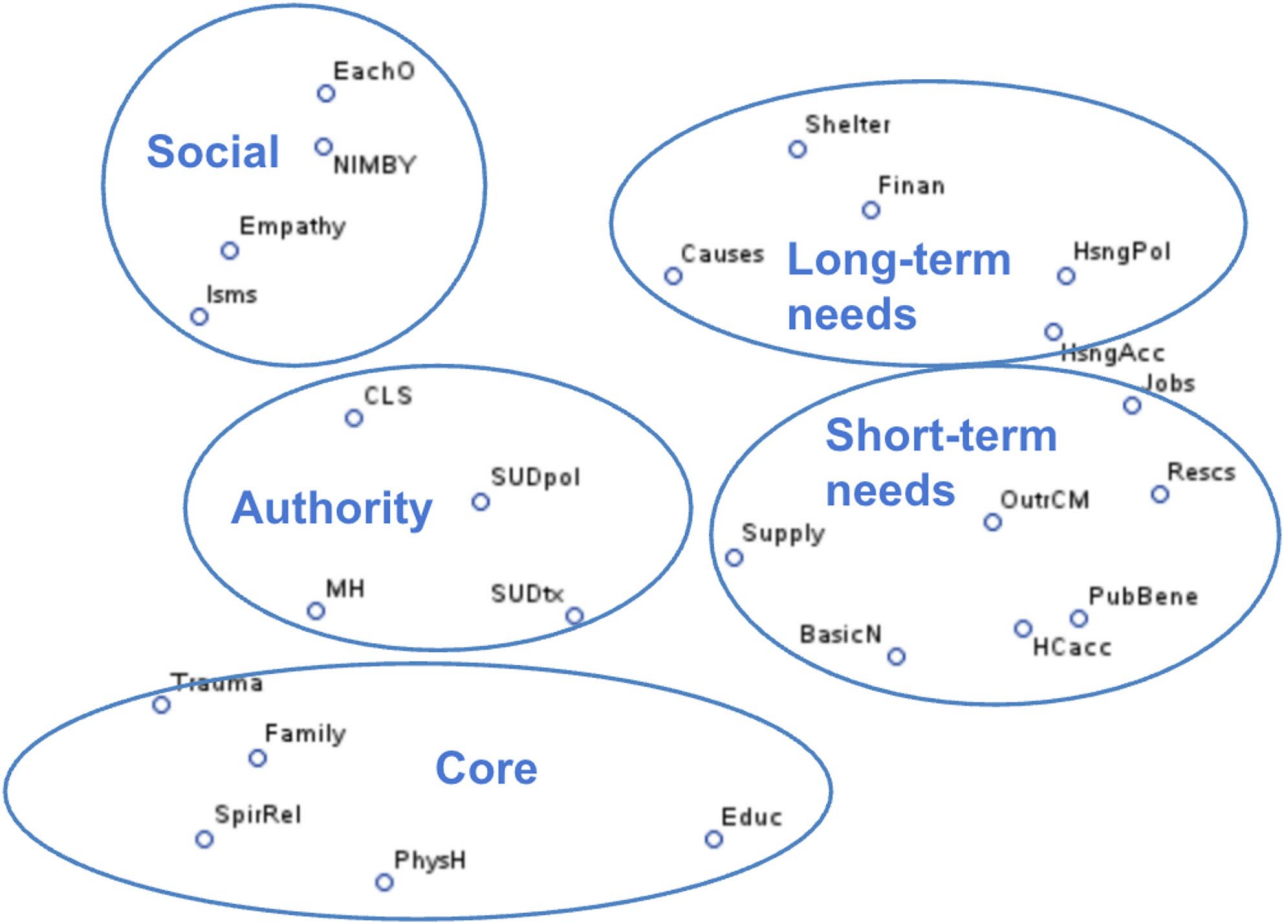
This is a plot of the 25 topic statements research partners sorted entitled “Labeled Clusters.” In the image there are circles around the five clusters. Starting from the top left and proceeding clockwise, there is a cluster titled “Social” that includes “Each other as a homeless community,” “Community reactions to homelessness,” “How to be empathetic and non-judgmental to people who are homeless,” and “Effects of stereotyping, stigma, racism and other -isms,” a cluster titled “Long-term needs” that includes “Shelter policies and conditions,” “Poverty and financial instability,” “Housing policy (like rental restrictions, credit checks),” “Housing availability, affordability, and stability,” and “Causes of homelessness,” a cluster titled “Short-term needs” that includes “Unemployment and job opportunities,” “Available resources and programs,” “The need for more outreach workers and case managers,” “How to access public benefits (like SNAP, Medicaid, housing vouchers),” “Access to health care,” “How to access basic needs (like food, clothing, hygiene, restrooms, heat),” and “What supplies people need to survive in different weather (like cold, rain),” a cluster titled “Core” that includes “Effects of trauma,” “Family issues and support,” “Spirituality and religion,” “Health and physical well-being (like pain),” and “Educational needs,” and a cluster titled “Authority” that includes “Policing and courts,” “How to care for mental health conditions (like anxiety and depression),” “Substance use policy (like what is legal and illegal),” and “How to care for substance use and addiction”. Legend: MH: How to care for mental health conditions (like anxiety and depression). SUDtx: How to care for substance use and addiction. HsngAcc: Housing availability, affordability, and stability. Finan: Poverty and financial instability. Rescs: Available resources and programs. Causes: Causes of homelessness. Isms: Effects of stereotyping, stigma, racism and other -isms. Trauma: Effects of trauma. CLS: Policing and courts. Shelter: Shelter policies and conditions. NIMBY: Community reactions to homelessness. PhysH: Health and physical well-being (like pain). HCacc: Access to health care. Jobs: Unemployment and job opportunities. Family: Family issues and support. EachO: Each other as a homeless community. BasicN: How to access basic needs (like food, clothing, hygiene, restrooms, heat). Educ: Educational needs. SUDpol: Substance use policy (like what is legal and illegal). HsngPol: Housing policy (like rental restrictions, credit checks). SpirRel: Spirituality and religion. Supply: What supplies people need to survive in different weather (like cold, rain). Empathy: How to be empathetic and non-judgmental to people who are homeless. PubBene: How to access public benefits (like SNAP, Medicaid, housing vouchers). OutrCM: The need for more outreach workers and case managers

For the first step, the co-authors typed out each response verbatim, editing only typographically. After discussing these as a team and identifying the themes we saw, we then inputted this list into ChatGPT (v. 3.5) with the instruction, “Identify the twenty-five most commonly mentioned themes from these responses.” We took this step as a check against our own biases, as the co-authors hold counterhegemonic perspectives of homelessness due to lived and practice experience (we see the causes of homelessness as primarily structural while the social narrative is one of individual blame). We did so with the recognition that ChatGPT, like other generative artificial intelligence (AI) tools, transmits the biases of the sources from which it draws, which in turn reflect those of society and the scientific establishment. Here we found it useful to compare these two opposing directions of bias as a check against the unintentional imposition of external perspectives on partners’ data. In actuality, when we reviewed the list generated by ChatGPT we found it concordant with what we had identified. We made minor edits for clarity and used these topic statements in step two (see Fig. 1). The ethics of the use of AI are further discussed below.

For step two, the co-authors input the information from the worksheets into Google Sheets. For the sorting worksheet, we entered partners’ responses into a 25 × 25 matrix, adding a 1 in the corresponding squares if they had grouped the statements together. We summed these matrices, then subtracted each value from the total response number (50) to create a dissimiliarity matrix. For the ratings worksheet, we obtained average ratings for the individual topics and for the clusters (described below). We also numerically coded the demographic information.

The co-authors used the Statistical Analysis System (SAS) Studio (v. 3.8) to complete the multidimensional scaling (MDS) and hierarchical cluster analysis (HCA). MDS is a set of statistical techniques that visually represents the similarity (proximity) or dissimilarity (distance) among items. For example, if one input a dissimilarity matrix with flight distances between major US cities, the resulting MDS plot would approximate a map of the United States. When used with conceptual data, topics that partners more frequently group together appear closer together. HCA is a method of exploratory data analysis used to identify groupings within a dataset. We generated HCAs using two methods and visualized six of the clustering solutions by circling these on the MDS plots. Finally, the co-authors discussed which solutions had the most conceptual validity and reached consensus on a five-cluster solution. During this conversation, the co-author with lived experience with homelessness highlighted conceptual connections that were not initially apparent to the other two co-authors. For example, there was some initial confusion about the conceptual coherence of the cluster that included the items *policing and courts*, *substance use policy*, *how to care for substance use and addiction*, and *how to care for mental health conditions*. The co-author with lived experience explained that for many people experiencing homelessness, the only behavioral health treatment they have received is that which was mandated by the courts in the context of substance-related arrests and convictions. The co-authors discussed this postulate together and reached consensus that it did best explain this grouping of topics. This finding is further discussed in subsequent sections.

### Ethical considerations

The co-authors anticipated and intentionally managed ethical considerations related to dual relationships, privacy, retraumatization, financial coercion, ownership of outputs, and the use of AI. With respect to dual relationships, two of the three co-authors have existing connections with the community with which we partnered for the study. We managed this by acknowledging this fact with potential partners and saying explicitly that their decision to participate or not would not impact their connections to us or with any current or future resources. We also let partners know that we would not mention anything said in the research context in subsequent conversations unless that person were to initiate the discussion about it. In actuality, no partners expressed concern about prior connections with the co-authors, although they regularly acknowledged them.

To ensure partners’ privacy, we offered the option to do the study in a location of their choosing, including a private room at a local library or one of the coauthors’ cars. While a few partners opted for one of these settings, the vast majority preferred to do the study activity in community with peers, often in the encampments. This frequently looked like groups of four to ten people doing the study activities either in parallel or in collaboration with one another. As noted above, we welcomed this collective approach and found it to be a strong net positive.

When considering retraumatization, we recognized that while the study did not explicitly ask about sensitive topics, there was the potential that it could bring up triggering subjects. We included language acknowledging this in the consent script, and offered a community resource sheet. No partners reported or demonstrated distress, and we wonder if their decisions to largely complete the study activities in community mitigated any distress. We also recognize that it is possible someone may have experienced but not reported distress, particularly in the case of partners who completed the study activity with a peer researcher.

The ethics of payment for research activities is the subject of active debate, with dueling assertions that both overpayment and underpayment are potentially coercive [48]. We selected an amount roughly commensurate with the living wage in the study area, and we paid partners immediately following the consent process to minimize any sense that they had to perform a certain way in order to receive compensation. We also found this to be a more respectful and dignified way to approach payment, in keeping with our overarching perspective of partners as valued collaborators.

Especially in the case of CBPR, where the community is highly invested in the research process, who owns the outputs is an important consideration. Often the outputs that are useful to the community are significantly different than those expected by academic collaborators [49]. In this case, the co-authors produced an outline with bullet points describing each step of the research process, which was then adapted as a conference presentation and an academic manuscript, and is in the process of being adapted into an infographic for community dissemination. This approach aims to make the outputs readily accessible to both the local and academic communities.

Finally, the use of AI bears consideration. On the one hand, AI represents a continuation of the use of technology in qualitative research: programs such as NVivo are commonly used to assist with data analysis [50]. However, AI has the unique potential to do the analysis “for” and not “with” researchers, posing concerns about intellectual integrity [51] and algorithmic bias [52]. Sensitive to these concerns, we used it much like one would use an additional coder: as a point of reference to check our inter-rater reliability and explore any discrepancies. Usefully in this context, we anticipated that AI would have a bias opposite ours given social perceptions of homelessness. In practice, the topic statements that we generated and which AI generated were substantively similar, so this check was primarily confirmatory.

## Results

Step one resulted in 257 statements from the 43 partners. These ranged from the specific and concrete, such as “CES [Coordinated Entry System] referral process – how hard it is to access shelter,” “Lack of vouchers, landlords don’t want to take them, BCI [criminal background check] issues, warrants, felonies,” to the broad and philosophical, such as “Social misunderstanding, the stereotype,” and “Compassion.” As described above, we generated a list of 25 statements from these responses. These included *shelter policies and conditions*, *housing availability*,* affordability*,* and stability*, and *effects of stereotyping*,* stigma*,* racism and other -isms*. The full list is included with Fig. 1.

While by design we did not direct partners’ responses toward health care and social services (or toward any specific content area), four statements directly related to health care (*how to care for mental health conditions*, *how to care for substance use and addiction*, *health and physical well-being*, and *access to health care*) and seven statements directly related to social services (*housing availability*,* affordability*,* and stability*, *available resources and programs*, *shelter policies and conditions*, *educational needs*, and *how to access public benefits*,* the need for more outreach workers and case managers*). Numerous other statements also touched on these spheres less explicitly (e.g., *how to access basic needs*, *family issues and support*).

In step two, 50 partners sorted and rated these 25 statements. Table 1 shows partners’ demographics and experiences with homelessness. None are young adults and none are older adults. Gender is reflective of the state’s unsheltered population, of which 35% identifies as female [26]. White individuals are overrepresented, as people identifying as white only comprise 56% of the unsheltered population overall [53]. Partners are representative with respect to ethnicity: statewide 17% of unsheltered individuals identify as Hispanic/Latina/e/o [26]. A majority of partners (63%) were homeless for five years or longer at the time of the study. 84% reported having stayed in a place not meant for human habitation in the last month.

**Table 1:** Partner demographics and experiences with homelessness

Age	
25–39 years old 40–59 years old	65% 35%
Gender	
Female Male	33% 67%
Race	
White Black/other	83% 17%
Ethnicity	
Non-Hispanic Hispanic	82% 18%
Race-ethnicity	
White BIPOC	70% 30%
Time Homeless	
Less than 1 year 1–5 years 6–10 years More than 10 years	6% 31% 47% 16%
Places of stay	
* Any place not meant for human habitation* Tent Outside with nothing Couch Jail Hospital Vehicle Hotel Abandoned house Treatment Shelter	*84%* 81% 63% 48% 46% 38% 33% 33% 31% 27% 25%

Using the MDS procedure in SAS, we generated a plot (Fig. 1) representing how frequently partners grouped the statements together. The five-cluster solution we selected is represented by the circles. We developed a title for each cluster based on what the co-authors perceived to be the commonalities among its elements (see above for a more detailed description of these discussions). These clusters are: (1) connections to immediate and basic needs (short-term needs), (2) longer-term needs / factors for stability (long-term needs), (3) interactions with authority and compulsory services (authority), (4) core factors of well-being (core), and (5) the social experience of homelessness (social). Tables 2 and 3 show the average ratings of the clusters and the highest-rated individual elements. Partners rated immediate and longer-term needs highest, although there was not extensive variation among the cluster scores. The individual topic statements ranked most highly were those related to accessing housing, public benefits, behavioral health care, and basic needs. These are not concentrated in a single cluster. We found no statistically significant differences in cluster ratings by age, gender, or time homeless. Black, Indigenous, and People of Color (BIPOC) partners were more likely to rate the *authority* cluster as more important.

**Table 2:** Ratings of clusters

Cluster	Average rating
Connections to immediate and basic needs	3.66
Longer-term needs / factors for stability	3.48
Interactions with authority and compulsory services	3.45
Core factors of well-being	3.11
Social experience of homelessness	3.11

**Table 3:** Ratings of individual elements (Top 5)

Element	Cluster in which it is grouped	Average rating
Housing availability, affordability, and stability	Longer-term needs / factors for stability	3.93
Accessing public benefits	Connections to immediate and basic needs	3.78
Caring for substance use and addiction	Interactions with authority and compulsory services	3.77
Caring for mental health conditions	Interactions with authority and compulsory services	3.76
Accessing basic needs	Connections to immediate and basic needs	3.73

Partners’ free responses to what they saw as most important included: “Housing, we need more housing for the homeless…fix up all ‘The Bandos’ [abandoned houses] and get us out of the cold,” “How to care for substance use and addiction if I wasn’t using like this I wouldn’t be homeless like this. I still want to get high but I don’t want to be homeless,” and “Effects of trauma, because it affects almost every single category and the effect they have one’s self.” As with the topic statements, many of these open responses also spoke directly or indirectly to the relevance of social services and health care to addressing homelessness.

## Discussion

Partners saw homelessness and ways of addressing it as complex and multifaceted. This is reflected in the extent and scope of their responses to the initial prompt, which included descriptions of both specific details and broad social phenomena. As represented by the cluster ratings, partners prioritized immediate needs first, but not to the exclusion of longer-term, social, and existential needs. That housing availability, affordability, and stability was the highest-rated single item highlights that homelessness is at its core about lack of housing, while the other highly rated topic statements underscore its interconnection with other social structures, including the (dis)functionality of the social safety net and behavioral healthcare system.

In significant part, this aligns with the existing literature on the self-identified needs of people experiencing homelessness, which recognizes at least components of the five clusters: the importance of accessing food, clothing, and hygiene facilities [21] as part of immediate and basic needs; housing [27] as part of longer-term needs and factors for stability; trauma [35] as part of core factors of well-being; and negative community responses to homelessness and a lack of empathetic and non-judgmental reactions to people who are homeless as part of social experiences of homelessness [34].

However, visualizing the elements’ proximity to one another and groupings clarified how partners saw their relationships. For example, the literature highlights employment as both a way to meet one’s basic needs and to give the worker a sense of purpose [29]. That participants grouped it in the *short-term needs* cluster rather than the *core* cluster suggests that it is primarily viewed in practical terms. This is in contrast to the education item, which is located in the *core* cluster (and notably not in the *long-term needs* cluster). In keeping with prior studies that described behavioral health treatment as coercive and prescriptive [16], mental health and substance use treatment are in the *authority* cluster with policing and courts, rather than in the *short-term needs* cluster with access to health care and to outreach and case management or in the *core* cluster with health and physical well-being. This highlights how partners saw the route into behavioral health services as via compulsory rather than voluntary means.

This is salient given what the literature says about compulsory substance use treatment. A recent systematic review of involuntary treatment reported that only 22% of the included studies found positive results, with an equal percentage finding negative outcomes. The study’s authors conclude that the priority should be to develop voluntary treatment models [54]. One promising pathway to this is through primary care [55]. Another avenue is to connect people with behavioral healthcare with the ongoing support of a community health worker or other outreach worker [56].

This study contributes an informative and actionable typology of areas for intervention in the form of its clusters and ratings. While prior studies have identified many of the individual elements, their groupings provide a deeper conceptualization of how individuals see them as interrelated. This has practice and policy implications for healthcare and social services systems. In the case of the location of substance use treatment relative to potential points of connection for behavioral health services, these findings reinforce how service provision can – and should – improve accessibility by pivoting away from involuntary, criminal legal system-based behavioral health interventions and toward connections via community-based care, strategies supported by recent literature.

More broadly, these interconnections highlight the importance of structural competency in service delivery and policy development. To construct viable interventions to address homelessness, it is important to build from the perspectives and priorities of people who are themselves experiencing it. Apart from the *results* of the study, the *process* is also significant for what it offers partners, researchers, and their communities. To partners, it provides a platform for their voices and a validation of their hard-earned lived expertise. It gives them lightly structured opportunities to talk with their peers about their perspectives, thereby building community connections. It also sparks interest in research and opens doors for partners to participate in subsequent studies as co-designers and co-authors: several partners have already reached out to express their interest in playing a role like that of the co-author with lived experience in future research. For researchers with lived experience, studies like this provide new avenues of connection with former peers while building new skillsets and professional capital, while for researcher-practitioners without firsthand knowledge it reinforces the importance of person-centered and community-grounded practice. For the community as a whole, it offers a tangible, feasible way to gather and visualize the understandings of those directly impacted, thereby offering a roadmap for further study and action. While the details of this roadmap are by design situated in a particular local context and thus not generalizable, the means by which it is developed are widely replicable and adaptable.

This study has several important limitations. Firstly, the vast majority of the partners were people experiencing *unsheltered* homelessness – we cannot assume that the perspectives of this community are the same as those experiencing sheltered homelessness or acute housing instability. Additionally, the partners were English-speaking, did not include youth or older adults, and disproportionately identified as white. It is reasonable to expect that the results would be different if this study were conducted with speakers of other languages, people at either end of the age spectrum, and people of color. Moreover, this study took place in one state, and primarily within the metropolitan area of that state. Furthermore, in listening to the partners complete the study activities, it was clear that people interpreted the statements very differently: while this room for personal interpretation is an intentional element of the research design, it also presents a limitation when analyzing the results. Finally, the framework of the study design – what was required to secure IRB approval and funding – was designed prior to the systematized inclusion of people with lived experience with homelessness, a deviation from the full implementation of CBPR.

There are numerous areas for further study suggested by the study results and limitations, all of which present opportunities to further integrate partners into the research process. Procedurally, the co-authors have identified local resources that compensate community members for their time to review and collaborate on study materials before IRB approval, which presents opportunities for formalized input into the early stages of subsequent study design. Utilizing these resources to incorporate partners from inception, it would be valuable to plan subsequent community-based participatory research studies that further examine the highest-ranked individual topics and clusters. This could include using these methodologies to explore how people see access points to behavioral health treatments, what shifts they would like to see, and how these could be most functionally designed. It would also be informative to replicate this study design with other populations, including BIPOC people experiencing homelessness, youth, older adults, people who speak other languages, people in shelters and doubling up, people in rural areas, and people in other regions of the country or in other countries. Comparing the results of these studies could inform how people see the ecosystem of homelessness similarly and differently across demographic categories and social contexts. Finally, it would be valuable to replicate this study at subsequent time points, to see if practice and policy interventions (e.g., to expand voluntary access to substance use treatment) shifts community perceptions about these interrelations.

## Conclusions

This study confirms that community-based participatory research methods such as group concept mapping can be used effectively with people experiencing unsheltered homelessness to gather their perspectives on the homelessness crisis and to generate a roadmap for research and action. Beginning with community responses to an open-ended question, this multi-step process generated visualizations and ratings for how members of this community see the complex factors involved with homelessness and what is needed to effectively address the homelessness crisis. The identified clusters and their ratings – short-term needs (3.66), long-term needs (3.48), interactions with authority (3.45), core needs (3.11), and the social experience of homelessness (3.11) – are useful in conceptualizing how people experiencing homelessness see the topics within these areas as related to one another. Considering this together with what specific topic statements individuals rated most highly (housing access, accessing benefits, substance use and mental health care, and basic needs) reinforces much of the existing literature on the self-identified needs of people experiencing homelessness while adding nuance regarding partners’ perceptions of the interconnections among these elements.

These results have implications for healthcare and social services delivery and policy development, suggesting ways to adapt interventions to improve the accessibility of key resources, including voluntary, community-based linkages to behavioral health services. Equally importantly, by including people with lived experience as partners in multiple stages of the research process, these methodologies offer opportunities for validation, connection, and advancement for people with firsthand understanding while underscoring for other researchers the importance and feasibility of centering these perspectives. These processes have generalizability beyond the specific results.

## Data Availability

We have not yet publicly archived our data but are agreeable to doing so. Here https://docs.google.com/spreadsheets/d/1oo-qVh2AJN5K-RMbdI9IVzwL1UKL6aCbG0mDTqnsmqc/edit?usp=sharing is a viewable link.

## Declarations

### Authors’ information

As noted in the body of the text, MS is an early-career academic who has done homelessness outreach for almost two decades but who has not herself experienced homelessness, LG is a Master of Social Work student without personal experience with homelessness, and DA has recent personal experience with unsheltered homelessness.

## Abbreviations

CBPR: Community-based participatory research
GCM: Group concept mapping
SAS: Statistical Analysis System
MDS: Multidimensional scaling
HCA: Hierarchical cluster analysis
AI: Artificial intelligence
CES: Coordinated Entry System
BIPOC: Black, Indigenous, and People of Color
